# Supplementary material for: Export of piRNA precursors by EJC triggers assembly of cytoplasmic Yb-body in Drosophila
Source: Nat Commun. 2016 Dec 8;7:13739. doi: 10.1038/ncomms13739 (PMC5155165; doi:10.1038/ncomms13739)
Supplement: Supplementary Information — Supplementary Figures and Supplementary Tables [file ncomms13739-s1.pdf]

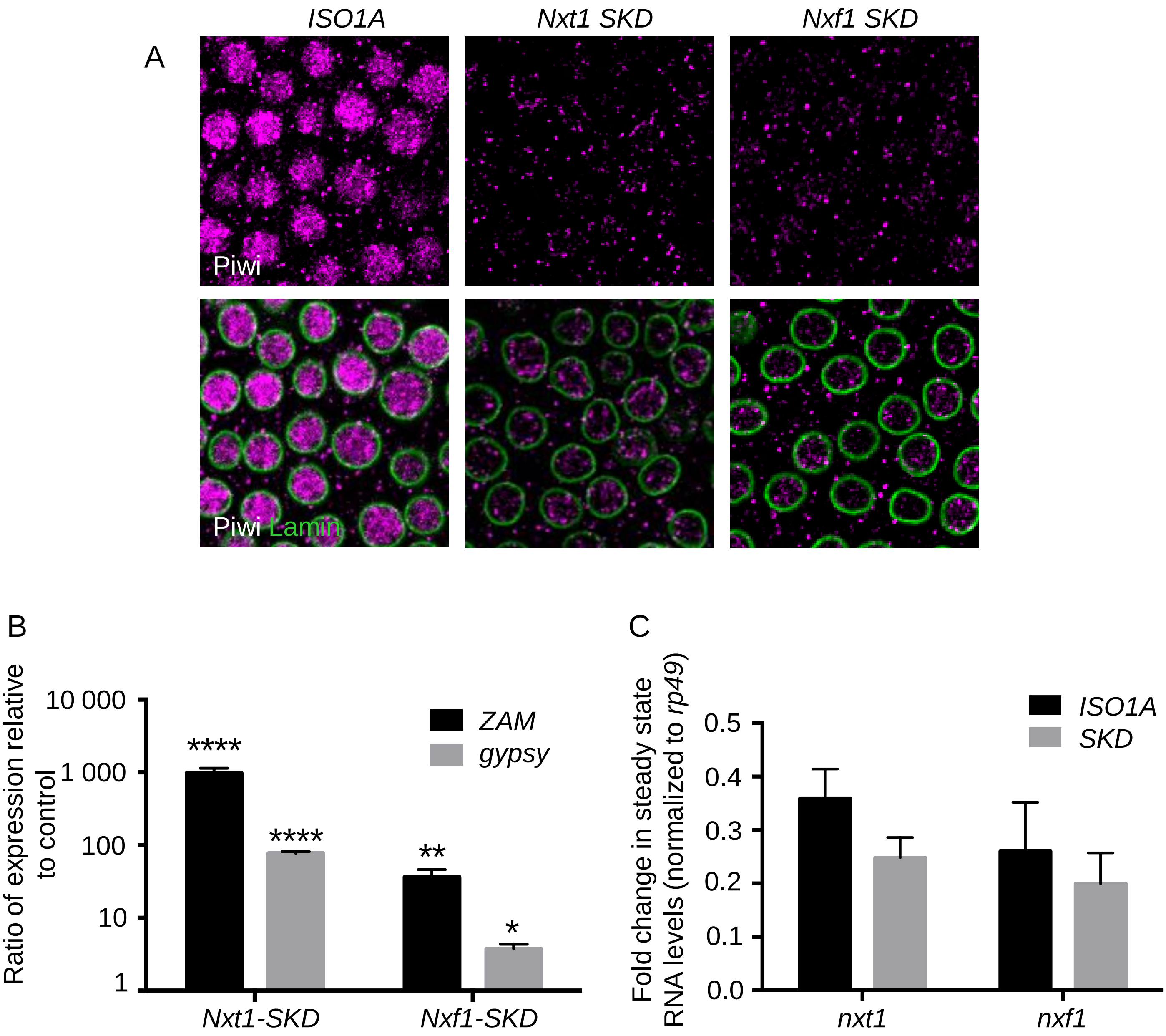

Supplementary Figure 1: *Nxt1* and *Nxf1* somatic knock-downs cause loss of Piwi nuclear localization and de-repression of transposable elements

(A) Piwi (magenta) and nuclear membrane (green) are visualized by immunofluorescence using anti-Piwi and anti-lamin antibodies, respectively, in *ISO1A* and *Nxt1*- and *Nxf1-SKD* lines. (B) Ratio of relative expression of endogenous *ZAM* and *Gypsy* transposable elements in ovaries from *Nxt1*- or *Nxf1-SKD* to control *ISO1A*. RNA levels are normalized to *rp49* level. Data are presented as means (n=3). Error bars indicate SEM. These values were transformed in log ratio to do statistical analysis. (****) p-value < 0.0001, (**) p-value < 0.01, (*) p-value < 0.05 according to the Student's t-test. (C) Fold change in steady-state RNA levels of *nxt1* and *nxf1* in *ISO1A*, *Nxt1*- and *Nxf1-SKD* ovaries. RNA levels are normalized to *rp49* level. Data are presented as means (n=3). Error bars indicate SEM.

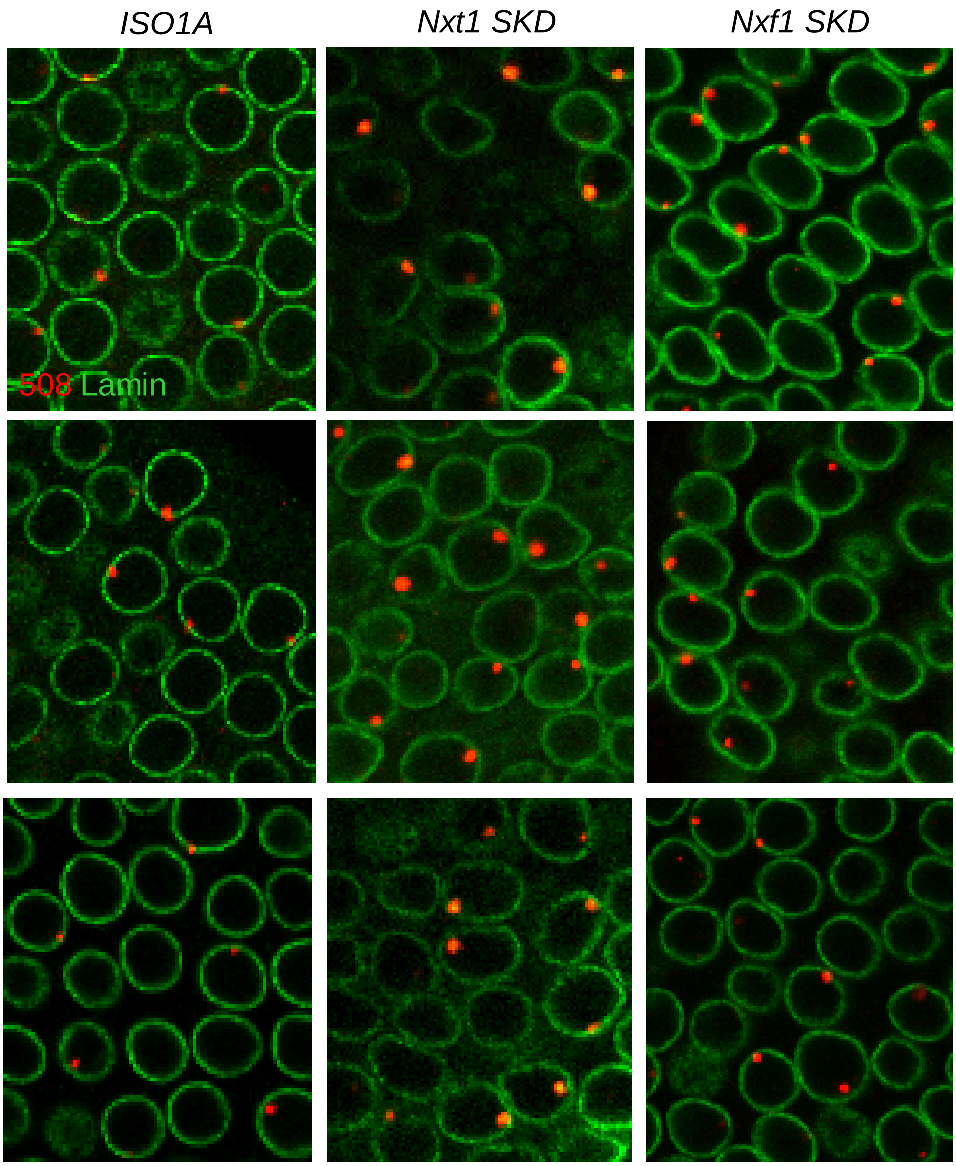

Supplementary Figure 2: Exportin Nxt1 and Nxf1 are implicated in *flam* RNA export

flam transcripts (red) and nuclear membrane (green) are visualized by RNA-FISH coupled to immuno-fluorescence using *flam* 508 RNA probe and anti-lamin antibody in *ISO1A* (left), *Nxt1*- (middle) and *Nxf1-SKD* (right) lines respectively. For each line three independent fields are shown. One Z-stack of confocal image is shown for each field.

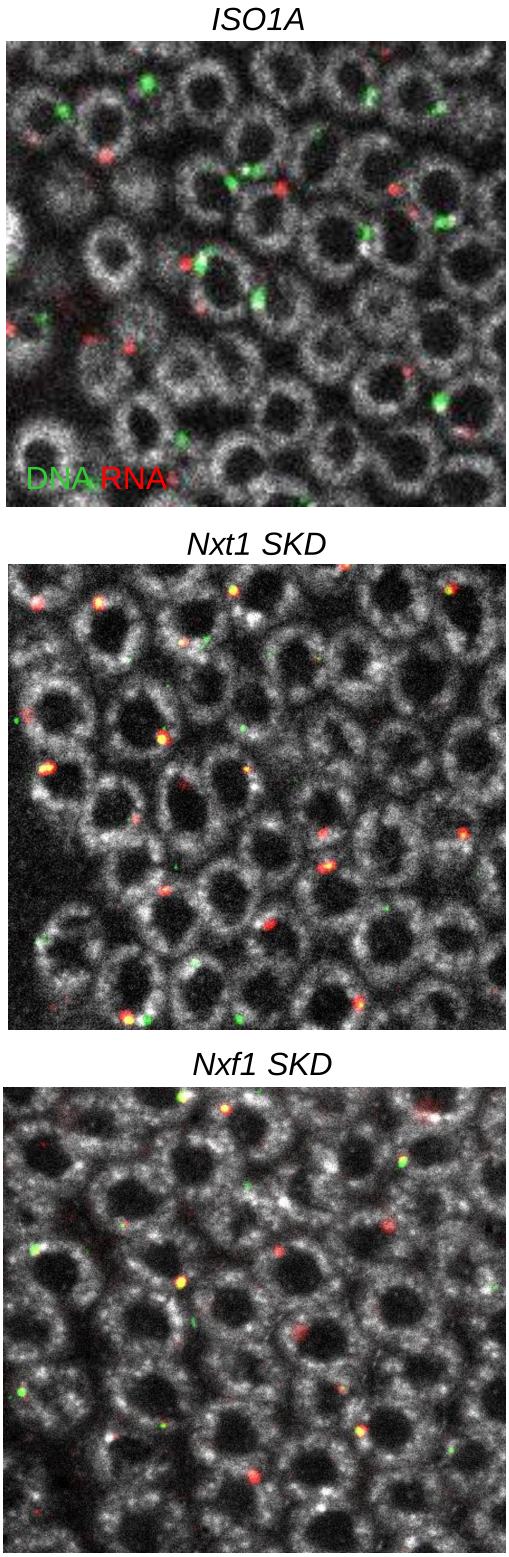

Supplementary Figure 3: Exportin Nxt1 and Nxf1 are implicated in *flam* intra-nuclear traffic to Dot COM

Double DNA/RNA FISH experiments performed on *ISO1A* ovaries (top), *Nxt1-* (middle) and *Nxf1-SKD* (bottom) lines. *flam* DNA (green) and *flam* transcripts (red) are respectively detected with DNA probe and 508 riboprobe. DNA is stained with Hoescht (white). A large field of one Z-stack of confocal image is shown for each line.

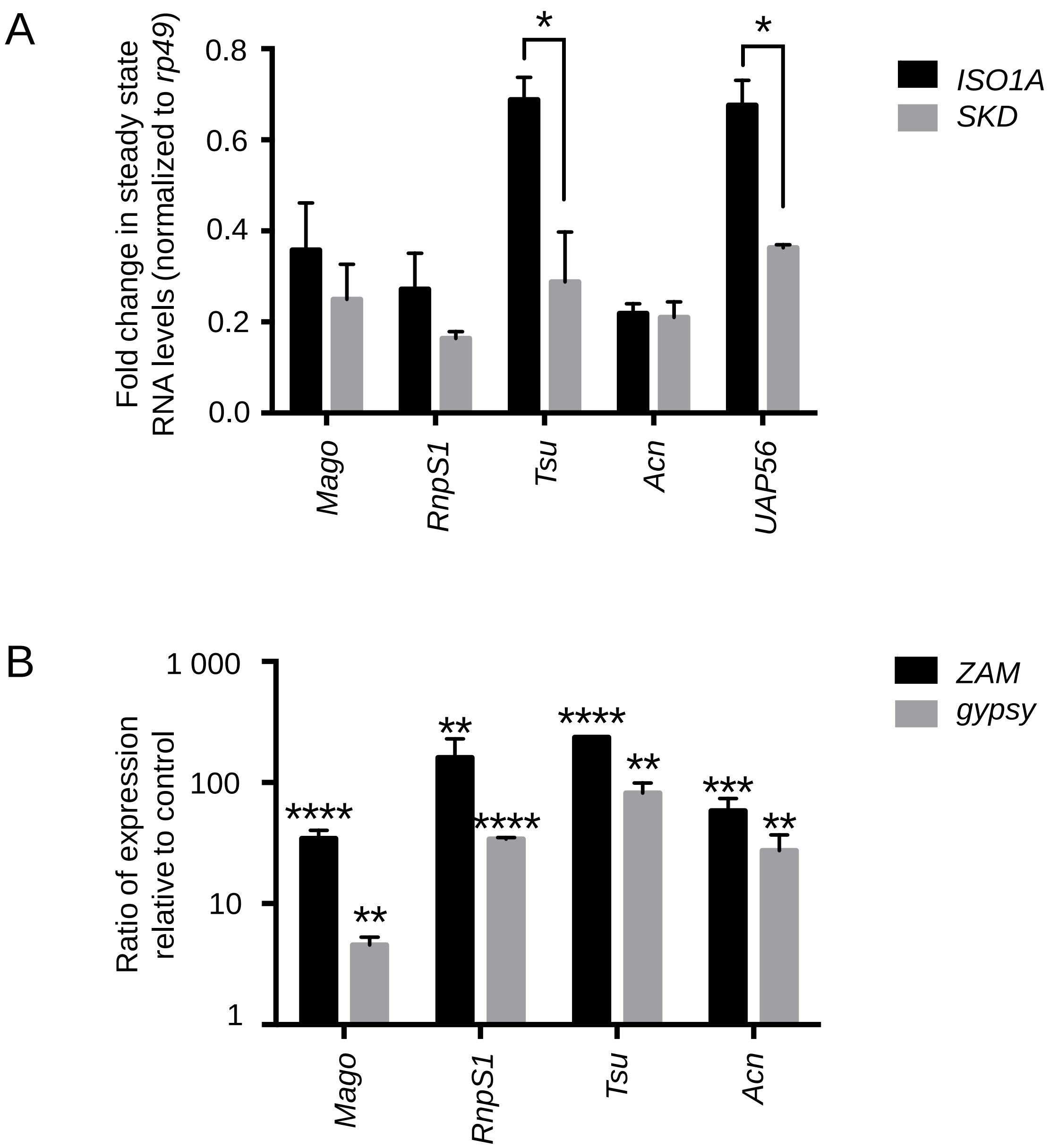

Supplementary Figure 4: *Mago-, RnpS1-, Tsu-, Acn-* and *UAP56*-somatic knock-downs cause de-repression of transposable elements

(A) Fold changes in steady-state RNA levels of *mago, rnpS1, tsu, acn* and *uap56* respectively in *Mago-, RnpS1-, Tsu-, Acn-* and *UAP56- SKD* ovaries. RNA levels are normalized to *rp49* levels. Data are presented as means (n=3). Error bars indicate SEM. (*) p-value < 0.05 according to the Student's t-test. (B) Ratio of relative expression of endogenous *ZAM* and *gypsy* transposable elements in ovaries from *Mago-, Rnps1-, Tsu-,* and *Acn-SKD* to WT control (*ISO1A*). RNA levels are normalized to *rp49* level. Data are presented as means (n=3). Error bars indicate SEM. These values were transformed in log ratio to do statistical analysis. (****) p-value < 0.0001, (***) p-value <0,001, (**) p-value < 0.01 according to the Student's t-test.

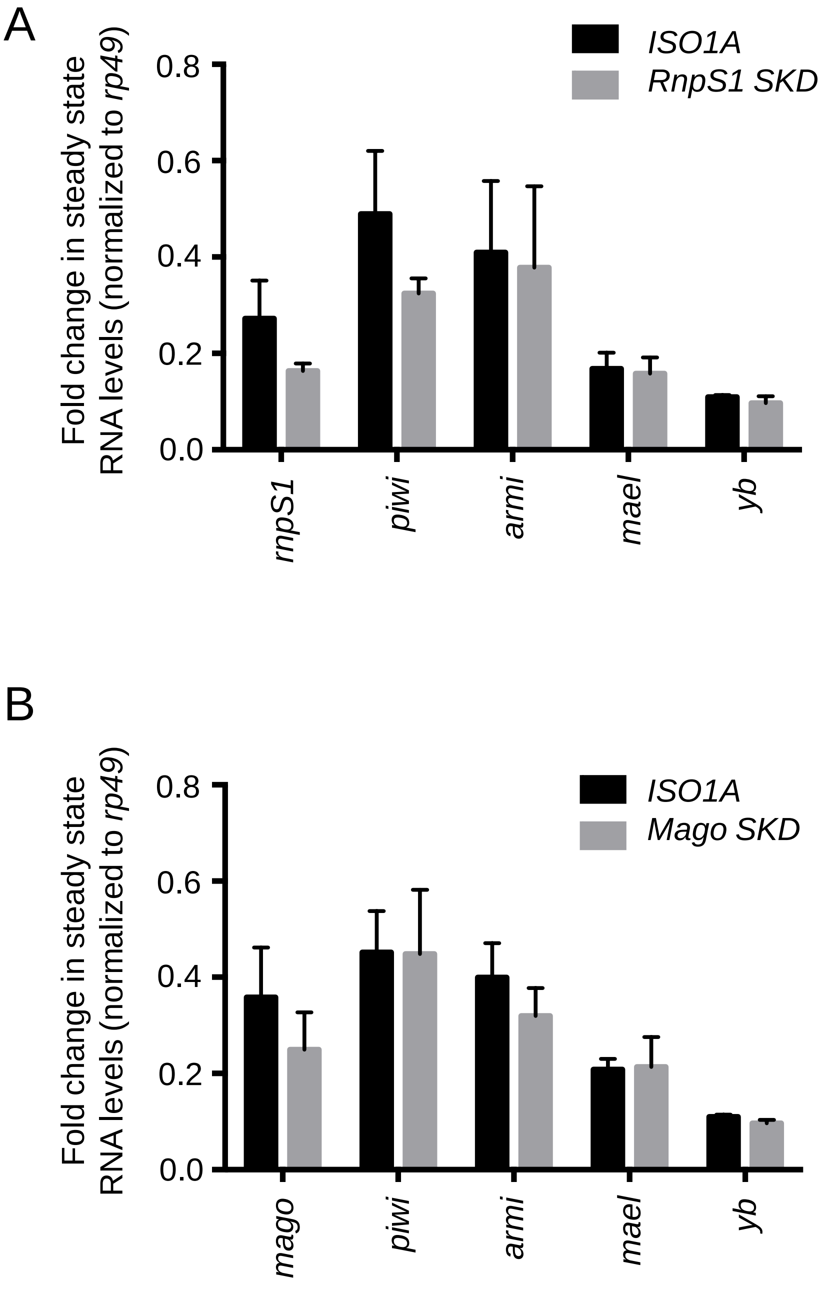

Supplementary Figure 5 : Levels of transcripts of piRNA pathway genes in *RnpS1*- and *Mago-SKD lines.*

Fold changes in steady-state RNA levels of *rnpS1*, *piwi*, *armi*, *mael* and *yb* in *Mago-SKD* line (A) and of *mago*, *piwi*, *armi*, *mael* and *yb* in *RnpS1-SKD* line (B). RNA levels are normalized to *rp49* levels. The sequence of the primers used are given in Supplementary Table 2. Data are presented as means (n=3). Error bars indicate SEM.

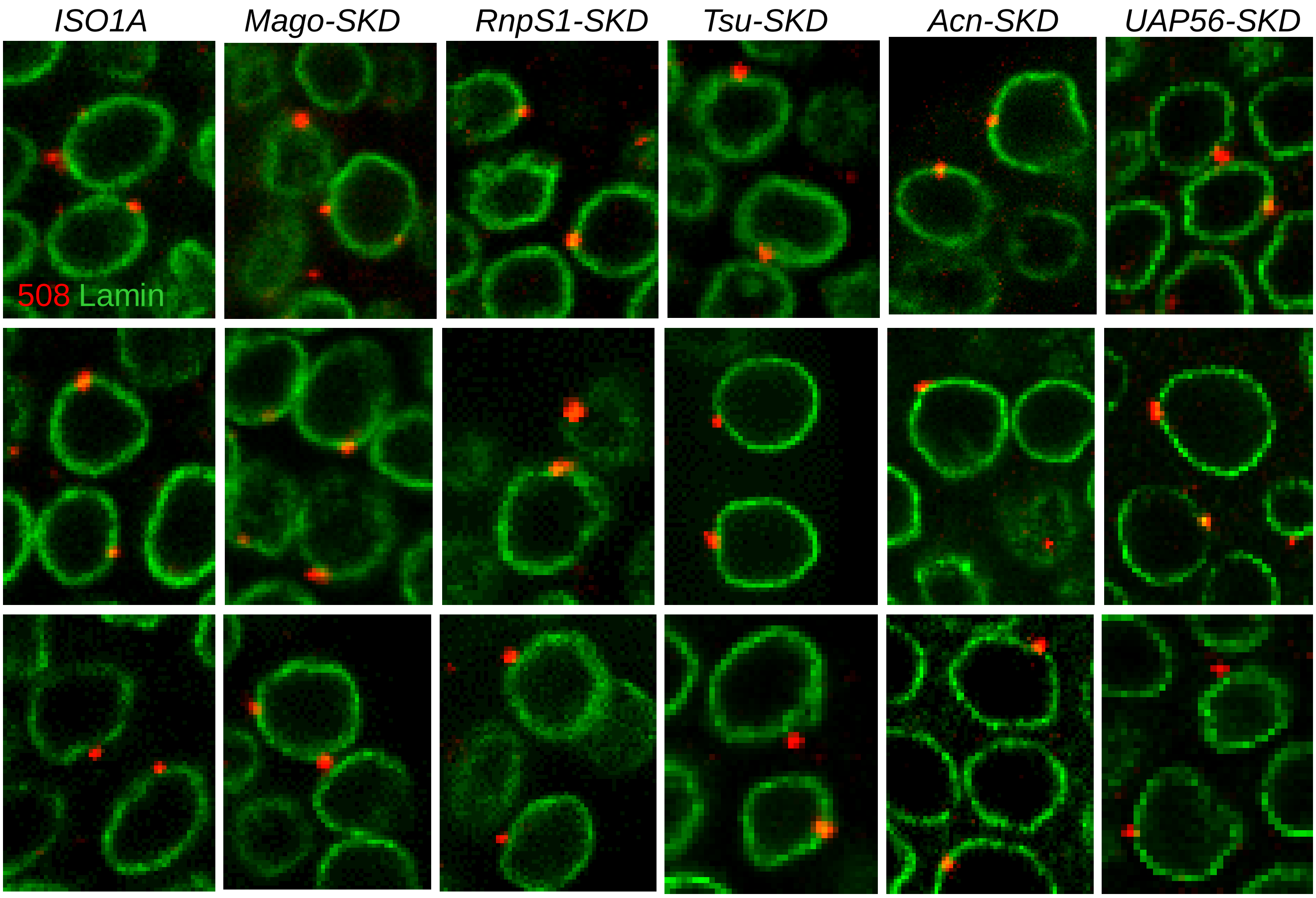

Supplementary Figure 6: *flam* transcripts are correctly exported in *EJC*- and *UAP56*-depleted lines

flam transcripts (red) and nuclear membrane (green) are visualized by RNA-FISH coupled to immunofluorescence using *flam* 508 RNA probe and anti-lamin antibody, respectively, in ovarian follicle cells of WT *ISO1A* and *Mago*-, *RnpS1*-, *Tsu*-, *Acn*- and *UAP56-SKD* lines. For each line three independent fields are shown. One Z-stack of confocal image is shown for each field.

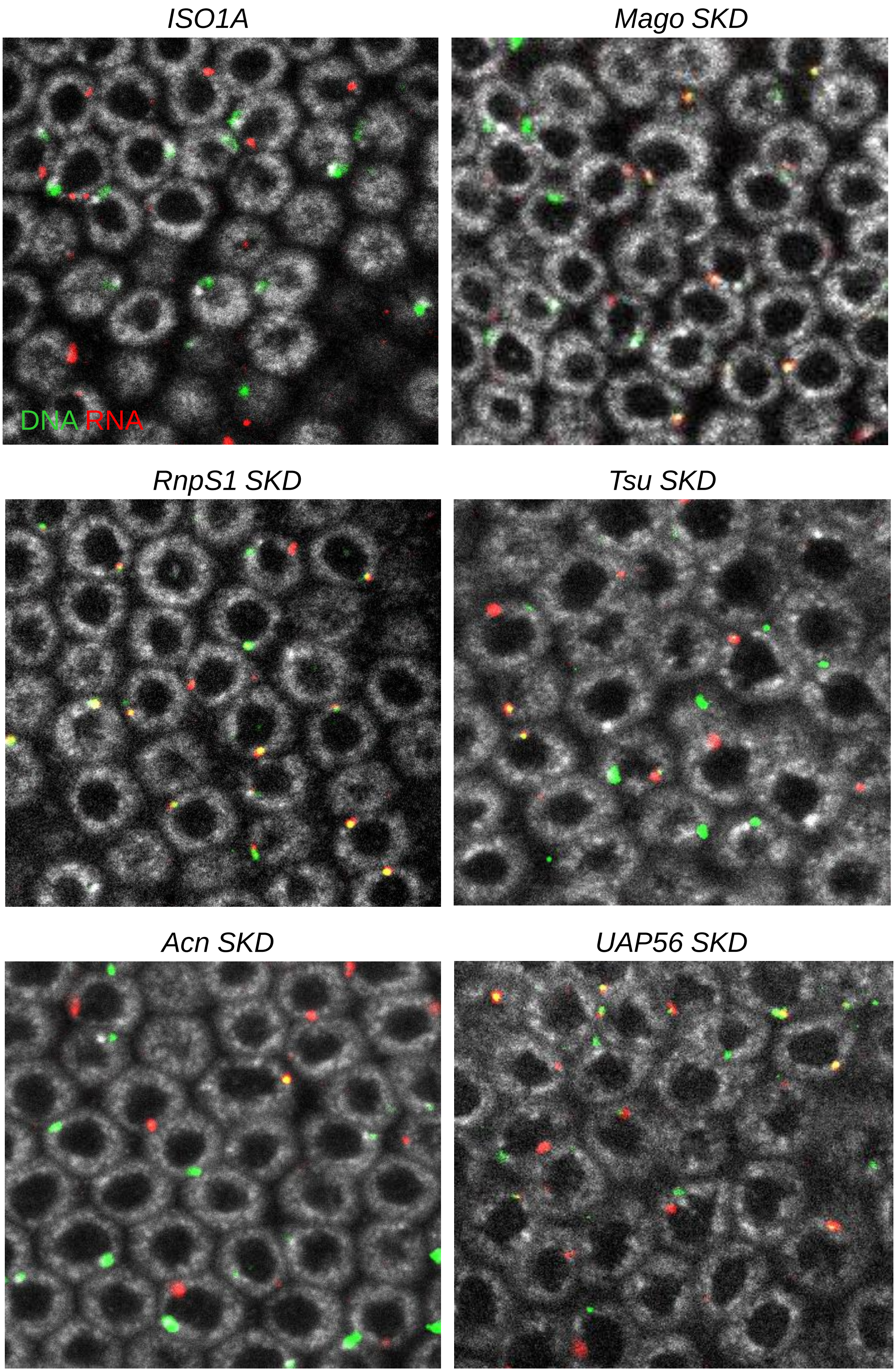

Supplementary Figure 7: Components of EJC and UAP56 protein are involved in *flam* transcript intra-nuclear traffic to Dot COM

flam DNA (green) and *flam* transcripts (red) are visualized by Double DNA/RNA FISH experiments in ovarian follicle cells of WT *ISO1A* and *Mago*-, *RnpS1*-, *Tsu*-, *Acn*- and *UAP56-SKD* lines. DNA is stained with Hoescht (white). Large fields of one Z-stack of confocal image are presented.

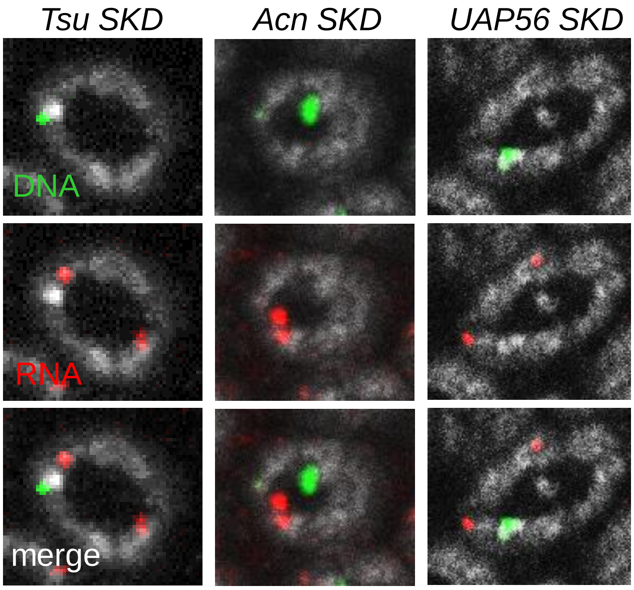

Supplementary Figure 8: Components of EJC and UAP56 protein are involved in the gathering of *flam* transcripts to a single focus

flam DNA (green) and *flam* transcripts (red) are visualized by Double DNA/RNA FISH experiments in ovarian follicle cells of *Tsu*-, *Acn*- and *UAP56*-depleted lines. Two foci of *flam* transcripts accumulate away from the *flam* piRNA cluster. DNA is stained with Hoescht (white).

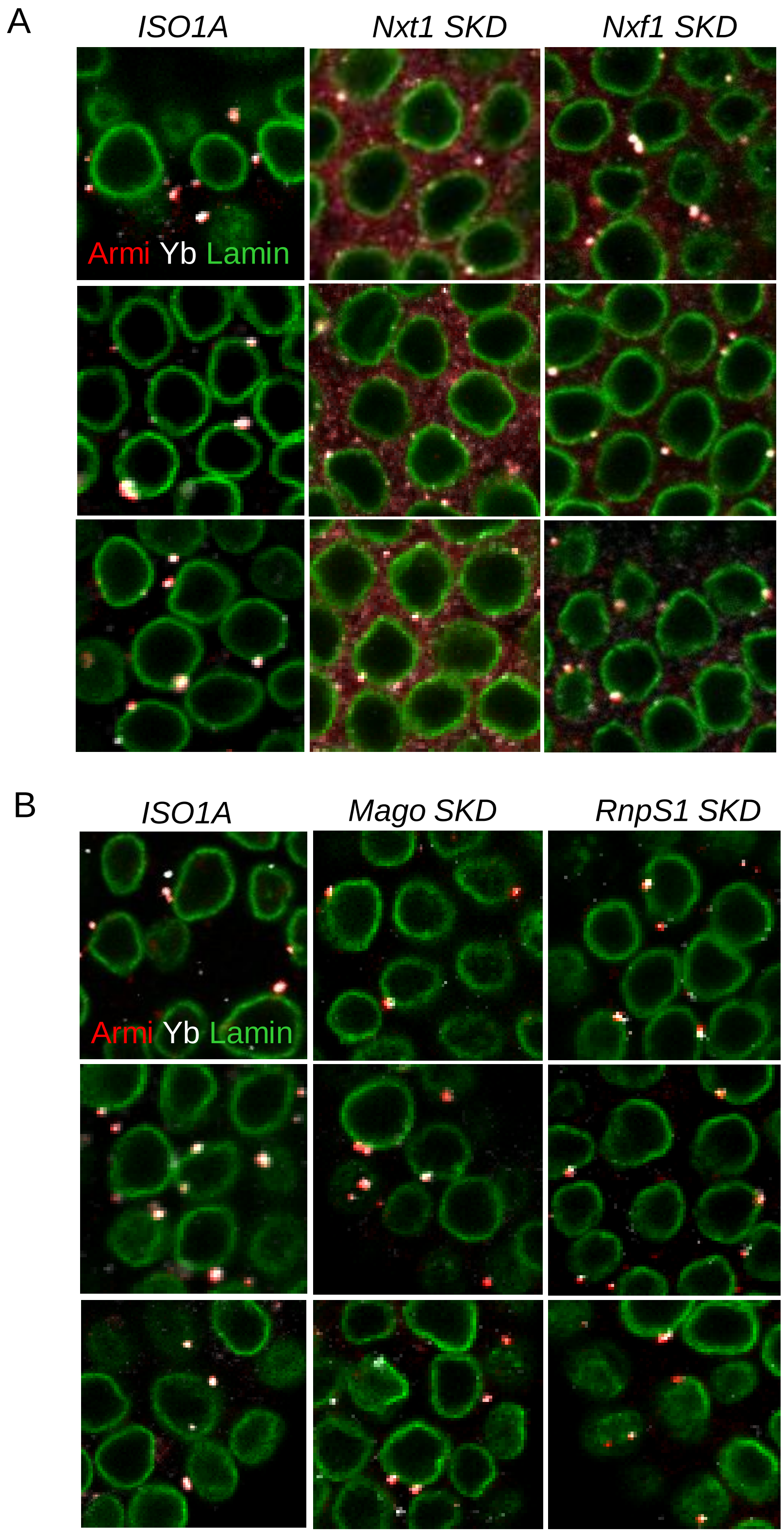

Supplementary Figure 9: Yb-body formation is altered in *Nxt1*- and *Nxf1*- but not in *EJC*-depleted follicle cells

Armi (red), Yb (white) and nuclear membrane (green) are visualized by immunofluorescence in *ISO1A* and *Nxt1*- and *Nxf1-SKD* follicle cells (A), and in *ISO1A* and *Mago*- and *RnpS1*-depleted cells (B). For each line three independent fields are shown.

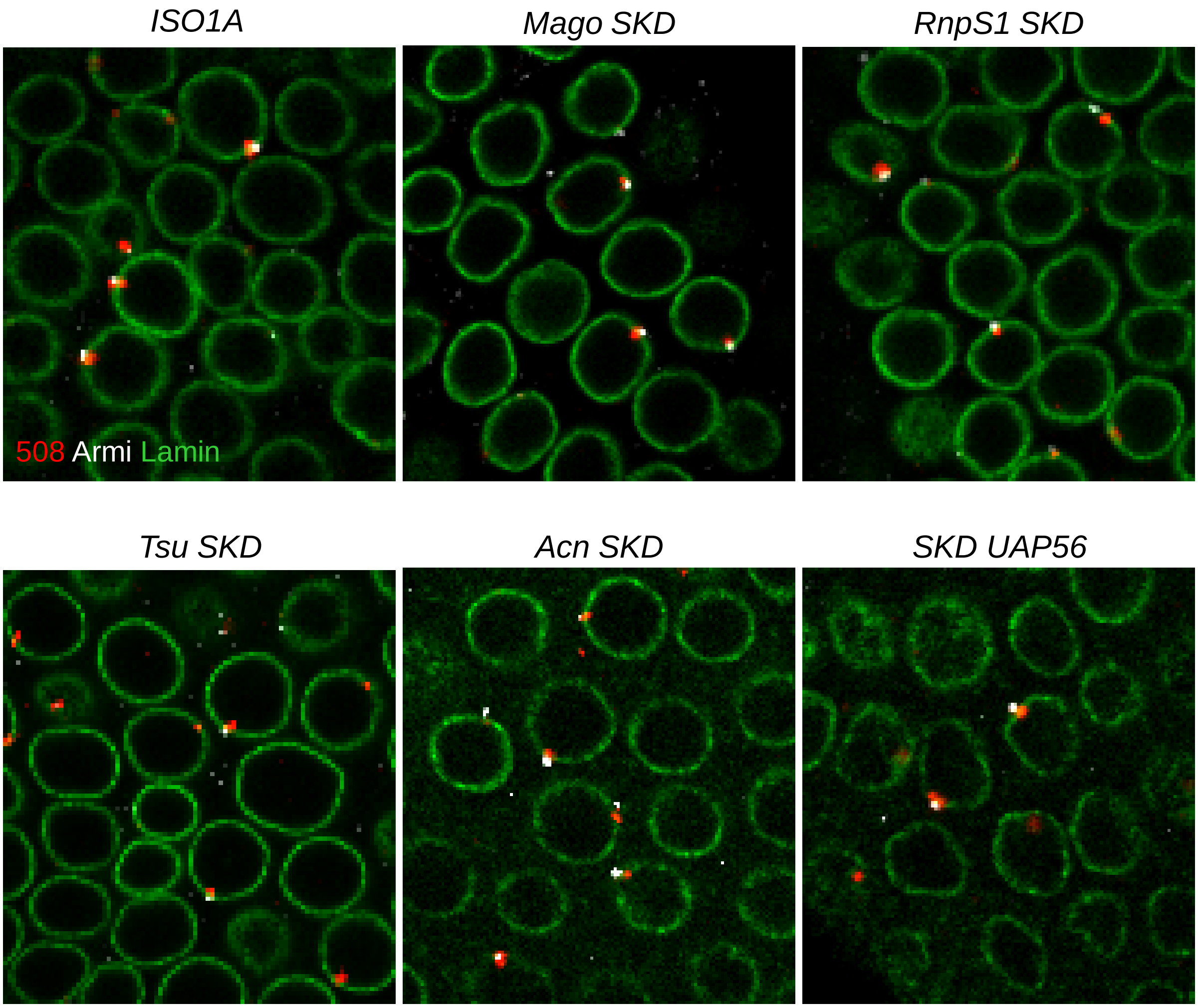

Supplementary Figure 10: Yb-bodies are assembled at the site of export of *flam* transcripts in *EJC*-depleted lines

flam transcripts (red), Armi foci (white) and nuclear membrane (green) are visualized by RNA-FISH coupled to immunofluorescence in ovarian follicle cells of *ISO1A* and *Mago*-, *RnpS1*-, *Tsu*-, *Acn*- and *UAP56*-*SKD* lines. A large field of one Z-stack of confocal image is shown for each line.

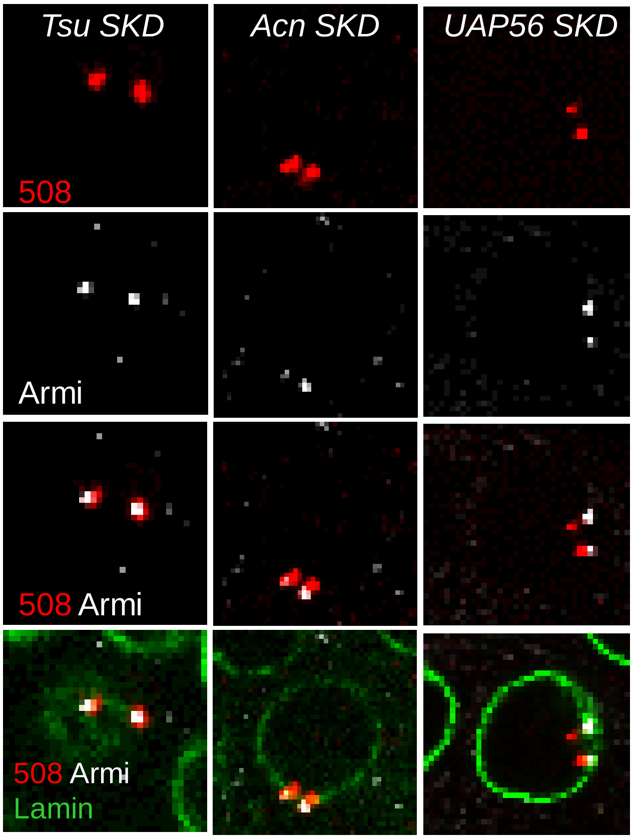

Supplementary Figure 11: Yb-bodies are juxtaposed to each site of export of *flam* transcripts in *EJC*-depleted follicle cells

RNA-FISH coupled to immunofluorescence in ovarian follicle cells staining *flam* transcripts (red), Armi foci (white) and nuclear membrane (green) in *Tsu*-, *Acn- and UAP56-SKD* lines show two Yb-bodies adjacent to two foci of accumulation of *flam* transcripts.

Position	Primers	
X:21,625,120.. 21,626,056	gtaagagctgtcccaaatacaa	acctacagcgacaccactta
X:21,628,276.. 21,629,208	tctgcccttccataagaaac	agcgaaaagttaactcacca
X:21,629,175.. 21,630,107	gcaaagtccggttaacaaat	ctttctgtgacctgagcagt
X:21,630,267.. 21,631,180	gcaaagtgatggaaagacg	ccttaagggactatgcctgt
X:21,631,376.. 21,632,292	gtttcgctgcttaagtcaca	gtacaaaatcttgggtgacg
X:21,632,351..21,633,313	tatgggacattggagatttg	cttagtcgaaccaatgttgc
X:21,633,322.. 21,634,202	catttctgtgcaacattgg	cactgcgaatcacctttaat
X:21,634,490.. 21,635,358	catttacggttttgtgaagg	tatctcgcgtcaaagaagag
X:21,635,450.. 21,636,301	gccaacgttgttgtttcat	caaagtcttgggtcaactgat
X:21,637,886.. 21,638,875	attctcctttctcaggatgc	gcattgctaccttacgtttc
X:21,638,747.. 21,639,707	agggaaaatgttacccgtta	ctctggaccaaactggatct

Supplementary Table 1: List of primers used to PCR amplify genomic fragments used as a DNA probe

Positions of amplified DNA were determined by mapping genomic positions to the Release 6.12 assembly. Primers are indicated in 5’ to 3’ orientation.

Gene Name	Primer names	Sequence 5'-3'
flam	exon2.1-F	TTATTTCTATGCCGGTTTGC
	exon2.1-R	GTTCGCTTGAAAGCTAGGAA
	exon2.2-F	AACATAATTATTAAAGGTGATTCGC
	exon2.2-R	GAAAGAGTGGGAGCGAGAGA
	exon3-F	TTGGCTATGAGGATCAGACA
	exon3-R	CTTCAAAGCGATTCATTCCT
gypsy	gypsy-F	GTTGAGGCAAGGATTGGAAA
	gypsy-R	TAAGCAGGTCAGCACCCTCT
ZAM	ZAM-F	AGAGTGAGAATGAAGTGCAGA
	ZAM-R	GTTAAGGCGGTTTATGTATTGTAT
Nxt1	Nxt1-F	GAGAATCGCGGGAAACAT
	Nxt1-R	GTCCACGGAGGCGTAGTACA
Nxf1	Nxf1-F	CAGTAGCCAGCGGTACAACA
	Nxf1-R	ATTGGGAGGGCTTAAAGCTG
Mago	Mago-F	GGAGATCATGCAGGAGGAC
	Mago-R	GAGGTGGTGAACGAGATGTG
RnpS1	RnpS1-F	AATCGGCAATACGAAAATGG
	RnpS1-R	CCTTGCCGTCCTTCTCTTT
Tsu	Tsu-F	ATTGACAATGCGGAGGAGT
	Tsu-R	TTACTGTCGCTTCCAAATCC
Acn	Acn-F	ATCGGGCAATACTTTCAACG
	Acn-R	AACATCCCATTCGCGTACAG
UAP-56	UAP-56-F	CAGCAAGGAGTATGAGCGATT
	UAP-56-R	CTTGAGGGTCTCCTCGTCCT

Supplementary Table 2: Primers for qPCR analysis

Gene Name	Primer names	Sequence 5’-3’
Piwi	Piwi-F	AAGTTCCAGTGCGATCATCA
	Piwi-R	TCCCATGAACTCCGAACTCT
Armi	Armi-F	CGGTTCTATGCCAACCAAGT
	Armi-R	TGAATAGTGCTGCTCGATGG
Mael	Mael-F	TTTTTAAGAAAGACTTTTTCGAGTTT
	Mael-R	AGTGAAGGTATACGCCCATC
Yb	Yb-F	GCCAGATCACTATCCCAAAA
	Yb-R	CTCATCGGAAAGTTGAGGAA
rp49	rp49-F	GACGCTTCAAGGGACAGTATCTG
	rp49-R	AAACGCGGTTCTGCATGAG

Supplementary Table 2: Primers for qPCR analysis (continued)
